# Phosphate of Ammonia, and Its Employment in Gout and Rheumatism

**Published:** 1850-11

**Authors:** 


*Phosphate of Ammonia, and its employment in Gout and Rheumatism.*

Dr. Samuel Edwards, in a paper read before the *Westminster Medical Society*, after giving a history of this salt, and its mode of preparation, remarked with regard to its physiological effect, that "in the ordinary dose of ten grains it sometimes occasions a slight degree of nausea, accompanied with heat of the epigastrium ; immediately after which, if the surface be kept warm, it acts as a stimulating diaphoretic, and also a diuretic." The proximate cause of gout and rheumatism he considers in the light of "blood diseases, arising from morbific matter circulating in the blood, originally formed in the primary and secondary assimilating processes," and consequently disturbing the parts to which it is attracted. He considers the two as varieties of the same disease.

From a number of experiments Dr. Edwards had arrived at the conclusion that both gout and rheumatism had lithic acid for their essential cause; that it was supplied by the nitrogenized elements of the food as well as the changing tissues of the body; and confirmed the fact that uric acid existed in the blood. On the physiological action of this remedy in these diseases, Dr. Edwards says:

On being taken up into the system, and coming in contact with the uric acid, or urate of soda, it becomes decomposed, a phosphate of soda and urate of ammonia would be produced; thus exchanging a very insoluble for a very soluble salt; but this is not all, for Baron Liebig has shown that the phosphate of soda has remarkable effect upon uric acid, rendering it soluble with facility in water. By these means, therefore, the combined and free uric acid existing in the system in these diseases will be dissolved and capable of easy elimination by the kidneys. Dr. Edwards then remarked that he had used the phosphate of ammonia in almost every variety of gout and rheumatism, and almost always with the most beneficial effects. He had warded off attacks by its early employment. Before using it he generally prefaced it by a purgative of calomel and colocynth, or some other, and in acute articular rheumatism adopted the usual local and general antiphlogistic treatment. It had been found extremely useful in those cases bearing a resemblance to neuralgic disease. Another important fact to which Dr. Edwards drew attention was, that in fifteen cases in which he had used this remedy in *acute rheumatism*, in no one had *heart* symptoms accompanied it. He also described its solvent power as great in uric acid gravel, and asked, might it not be available in uric acid calculi? He had given it a comparative trial with phosphate of soda and benzoic acid, and found it far more useful in its effects. In some few cases of gout he had used a lotion of it, with good and soothing effects, especially where a concretion of urate of soda appeared to be forming.—*Dublin Medical Press, June*, 1850.—*From N. Y. Register of Med. and Pharmacy.*

